# Bidirectional relationship between *Helicobacter pylori* infection and nonalcoholic fatty liver disease: insights from a comprehensive meta-analysis

**DOI:** 10.3389/fnut.2024.1410543

**Published:** 2024-08-05

**Authors:** Daya Zhang, Qi Wang, Feihu Bai

**Affiliations:** ^1^Graduate School, Hainan Medical University, Haikou, China; ^2^Department of Gastroenterology, The Second Affiliated Hospital of Hainan Medical University, Haikou, China; ^3^The Gastroenterology Clinical Medical Center of Hainan Province, Haikou, China

**Keywords:** *Helicobacter pylori*, nonalcoholic fatty liver disease, meta-analysis, metabolic syndrome, incidence, risk factors

## Abstract

**Background:**

*Helicobacter pylori* (*H. pylori*) infection and nonalcoholic fatty liver disease (NAFLD) represent significant concerns in global health. However, the precise relationship between *H. pylori* and NAFLD remains a subject of ongoing debate. This study endeavors to elucidate the association between *H. pylori* infection and the susceptibility to NAFLD. Furthermore, we aim to investigate the interplay among *H. pylori* infection, NAFLD, and metabolic syndrome (MetS).

**Methods:**

We conducted an extensive search of the PubMed, EMBASE, and Web of Science databases spanning from inception to January 2024. Our examination focused on rigorous studies investigating the correlation between *H. pylori* infection and NAFLD. Utilizing a random-effects model, we computed the pooled odds ratio (OR) and corresponding 95% confidence interval (CI). Additionally, we assessed statistical heterogeneity, performed sensitivity analyses, and scrutinized the potential for publication bias.

**Results:**

Thirty-four studies involving 175,575 individuals were included in our meta-analysis. Among these, 14 studies (involving 94,950 patients) demonstrated a higher incidence of NAFLD in *H. pylori* infection-positive individuals compared to *H. pylori* infection-negative individuals [RR = 1.17, 95% CI (1.10, 1.24), *Z* = 4.897, *P* < 0.001]. Seventeen studies (involving 74,928 patients) indicated a higher positive rate of *H. pylori* infection in patients with NAFLD compared to those without NAFLD [RR = 1.13, 95% CI (1.02, 1.24), *Z* = 2.395, *P* = 0.017]. Sensitivity analyses confirmed the robustness of these findings, and funnel plot analysis revealed no significant publication bias. Furthermore, we observed associations between *H. pylori* infection or NAFLD and various metabolic factors, including body mass index (BMI), blood pressure, lipids, liver function, and kidney function.

**Conclusion:**

Our meta-analysis presents evidence supporting a reciprocal relationship between *H. pylori* infection and the susceptibility to NAFLD. Nevertheless, additional investigations are warranted to bolster this correlation and unravel the underlying mechanisms involved.

## 1 Introduction

*Helicobacter pylori* (*H. pylori*) is a bacterium with a Gram-negative structure known for its colonization of the gastric epithelium. This bacterium has been linked to the development of peptic ulcers, gastric cancer, and gastric mucosa-associated lymphoid-tissue (MALT) lymphoma (1, 2). *H. pylori* infection is known to be one of the most common gastrointestinal infections in humans (1, 2). A survey conducted on family units in China revealed a prevalence rate of approximately 40.66% for *H. pylori*, with rates of 43.45% in adults and 20.55% in children and adolescents (3). Despite a global decline in the prevalence of *H. pylori* infection from 58.2% in the period of 1980–1990 to 43.1% in the period of 2011–2022 (4), it continues to pose a significant clinical and public health burden. Furthermore, beyond its correlation with gastric disorders, *H. pylori* infection has been associated with a range of extragastric conditions, including stroke, Alzheimer’s disease, and nonalcoholic fatty liver disease (NAFLD) (5).

NAFLD is a significant public health concern affecting approximately 25% of the global population (6). According to a study published in 2022, the global prevalence of NAFLD was found to be 32.4% (7). Additionally, there is a projection indicating that the prevalence of NAFLD is expected to rise to 56% over the next decade (8). NAFLD includes both simple steatosis (SS) and non-alcoholic steatohepatitis (NASH), with the latter having the potential to advance to cirrhosis and hepatocellular carcinoma (HCC). In addition, NAFLD is closely associated with various extrahepatic conditions, including cardiovascular disease, obesity, diabetes, and hyperuricemia (9). Consequently, tackling NAFLD is paramount. Despite continuous research endeavors, the exact causes and mechanisms underlying NAFLD remain incompletely understood.

Insulin resistance and metabolic syndromes (MetS) such as hypertension, obesity, dyslipidemia, and type 2 diabetes mellitus are well-established risk factors for NAFLD (10). The correlation between *H. pylori* infection and the susceptibility to NAFLD has been explored in numerous studies; however, the results have been inconclusive. The findings of Polyzos et al. (11) suggest that *H. pylori* infection is an independent risk factor for NAFLD progression. In contrast, a cross-sectional study found that *H. pylori* infection was not listed as a risk factor for NAFLD (12). Furthermore, another observational study found no association between *H. pylori* infection and NAFLD diagnosis in a central European cohort (13). To our knowledge, the number of studies evaluating the impact of *H. pylori* eradication on NAFLD is limited, and the results of these studies are inconsistent. Some studies have found that *H. pylori* eradication may play a role in reducing the risk of NAFLD (14). Another study evaluated 13 patients with biopsy-proven NAFLD and showed that eradication of *H. pylori* had no significant long-term effect on hepatic steatosis (15).

Considering the escalating worldwide prevalence of NAFLD and its significant clinical and economic ramifications, it becomes crucial to elucidate the possible detrimental impacts of *H. pylori* infection on the risk of NAFLD. Therefore, we undertook a recent meta-analysis to investigate the association between *H. pylori* infection and NAFLD.

## 2 Materials and methods

### 2.1 Registration

This study was registered on the PROSPERO with a registration number CRD42023488399.

### 2.2 Literature search

The correlation between *H. pylori* infection and NAFLD was investigated by accessing the following databases: CNKI, VIP, Wanfang, PubMed, and Web of Science. The search period encompassed the establishment of these databases up until January 2024. Subject terms used in the search included “*Helicobacter pylori*,” “*Helicobacter pylori* infection,” “*Helicobacter*,” “*H. pylori*,” “HP,” “Nonalcoholic fatty liver disease,” “Nonalcoholic steatohepatitis,” “NAFLD,” “NASH,” “NAFL,” and others. The search was confined to full-text articles, and language restrictions were not imposed.

### 2.3 Eligibility criteria

(i) The study population should include patients with a diagnosis of NAFLD and detectable *H. pylori* infection; (ii) the study methodology should clearly report the diagnosis of *H. pylori* infection and NAFLD; and (iii) the study outcomes should include the counts of patients positive and negative for *H. pylori* infection, both with and without NAFLD.

### 2.4 Exclusion criteria

(i) Studies that did not exclude individuals with heavy alcohol consumption (usually defined as < 20 g/day for women and < 30 g/day for men) or other competing chronic liver diseases (e.g., viral hepatitis, iron overload, and use of potentially hepatotoxic drugs); (ii) Laboratory and animal studies; studies in pediatric populations (< 18 years); (iii) reviews, case studies, survey analyses, conference abstracts, and irrelevant literature; and (iv) duplicates of published literature.

### 2.5 Data extraction

Two independent evaluators reviewed the titles, abstracts, and full text of the literature obtained from each database. They assessed the eligibility of each article based on the criteria stated above. In cases of disagreement, the original articles were reviewed again, and consensus was reached through discussion. Pertinent information was extracted from the screened literature, including details such as authors, year, country, study type, sample size, gender, age, *H. pylori* testing method, and NAFLD diagnostic method.

### 2.6 Diagnosis

*H. pylori* infection can be detected through either invasive methods, such as endoscopic biopsy, or noninvasive tests including serology, the 13C or 14C urea breath test, and fecal antigen test. NAFLD diagnosis can involve histology, ultrasonography, or surrogate markers like the hepatic steatosis index (HSI), NAFLD-liver fat score (NAFLD-LFS), and/or fatty liver index (FLI).

### 2.7 Study quality assessment

Two evaluators used the JBI scale to assess the quality of cross-sectional study literature in ten areas. These areas included the purpose of the study, selection of the population, sample characteristics, inclusion and exclusion criteria for the sample, credibility and validity of data collection, authenticity of the data, ethical considerations, correctness of the statistical methodology, accuracy of the findings, and elaboration of the study’s value. A score of 14 or higher was considered indicative of high-quality literature. The quality of cohort study literature was evaluated using the NOS score, which assessed eight aspects. These aspects included the selection of the exposed and non-exposed populations, the method of measuring exposure factors, whether the outcome of interest occurred before the intervention, comparability of the exposed and non-exposed groups, accuracy and unbiasedness of outcome assessment, whether the follow-up duration was sufficient, and the adequacy of the follow-up process. For case-control studies, the NOS score evaluated their quality based on several factors. These factors included the appropriateness of case identification, representativeness of cases, selection of controls, identification of controls, comparability of cases and controls, identification of exposure factors, method of identification of exposure factors, and non-response rate. Scores ranging from 1 to 3, 4 to 6, and 7 to 9 were used to evaluate the low, medium-high, and high quality of the literature, respectively.

### 2.8 Statistical analyses

The extracted data were subjected to meta-analysis utilizing STATA 16.0 software. The specific process was as follows: (i) Effect size selection: dichotomous variables were evaluated using relative risk (RR), while continuous variables were assessed through weighted mean difference (WMD). Their corresponding 95% confidence intervals (CI) were then computed. (ii) Heterogeneity test: taking *P*-value and *I*^2^ as criteria, when *P* > 0.1 and *I*^2^ ≤ 50%, heterogeneity was small, and a fixed effect model (Fixed Effect, FE) was used for analysis. When *P* ≤ 0.1 and *I*^2^ > 50%, heterogeneity was large, and a random effect model (Random Effect, RE) was used for analysis. (iii) Evaluation of publication bias: Funnel plots and egger tests were drawn for the literature on the main indicators to evaluate whether there was a possibility of publication bias. (iv) Sensitivity analysis: In the case of notable heterogeneity among the primary indicators’ studies, sensitivity analysis ought to be conducted, and efforts made to identify the root cause of such heterogeneity.

## 3 Results

### 3.1 Study characteristics

**FIGURE 1:**
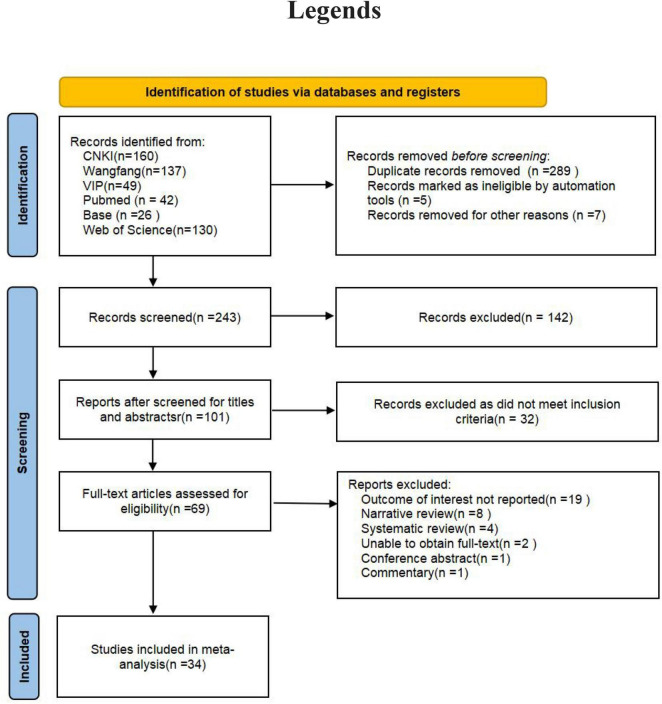
Flowchart of the study selection process.

The initial review identified a total of 544 documents. After removing duplicates and other unqualified documents, 243 studies were retained. Eventually, 34 studies were included in the analysis (Figure 1) (16–50). These 34 studies were published between 2011 and 2024. The majority of the studies were carried out in China, with the United States, Japan, Iran, Korea, and Brazil following suit. Study designs primarily consisted of cross-sectional, cohort, and case-control studies. In total, there were 175,575 patients included in the analysis. The distribution of male and female participants was approximately equal, and their ages ranged from 20 to 70 years. *H. pylori* infection was predominantly identified through the C13/C14 breath test, serum *H. pylori*-specific antibody assay, or urease test. The diagnosis of NAFLD was mainly established through abdominal ultrasound, abdominal CT, abdominal MRI, or liver biopsy. More detailed information can be found in Table 1.

**TABLE 1:** Characteristics of studies regarding the association of *H. pylori* infection with NAFLD.

References	Country	Type of study	Sample size	Gender		Age		Diagnosis of Hp infection	Diagnosis of NAFLD
				**Male**	**female**	**NAFLD/control**	**hp+/hp−**		
Chen et al. (16)	China	Cross-sectional study	962	529	433	–	–	^14^C-UBT	Abdominal ultrasound
Lisa et al. (17)	China	Case-control	504	306	198	41.91 ± 8.29/ 42.61 ± 8.17	–	^14^C-UBT	Abdominal ultrasound
Pazizge (18)	China	Case-control	2,323	1,010	1,313	46.25 ± 10.52/ 43.01 ± 14.30	–	^13^C/ ^14^C-UBT	Abdominal ultrasound
Shen (19)	China	Cross-sectional study	2,650	1,169	1,481	–	–	Serum Hp specific antibodies	Abdominal ultrasound
Wang (20)	China	Cross-sectional study	3,447	1,945	1,502	–	45 (36,53)/ 46 (36,54)	^13^C-UBT	Abdominal ultrasound
Zhen (21)	China	Cohort	2,063	1,091	972	51.38 ± 13.87/ 48.04 ± 15.08	48.36 ± 14.80/ 48.71 ± 15.03	^13^C-UBT	Abdominal ultrasound
Zhang (22)	China	Cross-sectional study	3,947	2,022	1,925	50.63 ± 11.43/ 50.61 ± 12.38	–	^14^C-UBT	Abdominal ultrasound
Wang (23)	China	Cohort	1,902	1,219	683	–	–	^13^C-UBT	Abdominal ultrasound
Xu and Li (24)	China	Case-control	1,000	565	435	53.43 ± 5.12/ 53.38 ± 5.06	–	^13^C-UBT	Abdominal ultrasound
Peng et al. (25)	China	Case-control	250	161	89	53.05 ± 9.62/ 53.45 ± 10.06	–	^13^C-UBT	Diagnostic criteria for non-alcoholic fatty liver disease
Wu et al. (26)	China	Case-control	114	–	–	–	45.6 ± 10.15/ 49.3 ± 10.45	^13^C-UBT	Abdominal ultrasound
Zhang and Ding (27)	China	Cross-sectional study	3,635	–	–	–	–	^13^C-UBT	Abdominal ultrasound
Xie and Yie (28)	China	Cohort	198	127	71	–	58.4 ± 1.4/ 57.1 ± 1.9	^14^C-UBT	Diagnostic criteria for non-alcoholic fatty liver disease
Yang et al. (29)	China	Cohort	160	78	82	–	52.378 ± 9.486/ 53.965 ± 6.685	^13^C-UBT	Abdominal ultrasound
Chen et al. (30)	China	Cohort	109	61	48	–	42.35 ± 12.34/ 42.65 ± 12.46	^13^C-UBT	Abdominal ultrasound
Wang et al. (31)	China	Cohort	848	441	407	–	59.49 ± 11.49/ 58.23 ± 12.15	^14^C-UBT	Abdominal ultrasound/ CT/ MRI
Guo (32)	China	Cross-sectional study	960	570	390	54.10 ± 12.72/ 52.02 ± 12.39	54.10 ± 12.72/ 52.02 ± 12.39	^14^C-UBT	Abdominal ultrasound
Zhang et al. (33)	China	Cross-sectional study	5,889	3,432	2,457	39.00 ± 15.00/ 32.00 ± 11.00	34.00 ± 15.0/ 33.00 ± 13.0	^13^C-UBT	Abdominal ultrasound
Liu (34)	China	Cross-sectional study	2,026	1,284	742	45.75 ± 11.25/ 42.50 ± 12.31	–	^14^C-UBT	Abdominal ultrasound
Liu et al. (35)	China	Cross-sectional study	14,373	9,164	5,209	50.32 ± 13.79/ 49.88 ± 14.00	–	^13^C-UBT	Abdominal ultrasound
Dong (36)	China	Case-control	296	163	133	46.18 ± 14.85/ 43.18 ± 14.46	–	Rapid Urease Test	Diagnostic criteria for non-alcoholic fatty liver disease
Shi et al. (37)	China	Cross-sectional study	5,000	2,990	2,010	50 ± 10/ 51 ± 9	–	^13^C-UBT	Abdominal ultrasound
Xu (38)	China	Case-control	112	60	52	44.2 ± 5.8/ 43.4 ± 6.2	–	Serum Hp specific antibodies	CT
Mohammadifard et al. (39)	China	Case-control	130	62	78	37.6 ± 5.6/ 36.6 ± 6.1	–	ELISA	Abdominal ultrasound
Jiang et al. (40)	China	Cross-sectional study	4,081	1,887	2,194	47.18 ± 13.33/ 42.37 ± 13.36	–	^13^C-UBT	Abdominal ultrasound
Wang et al. (41)	China	Cross-sectional study	71,633	30,086	41,547	–	46.5 ± 12.5/ 44.8 ± 13.3	^13^C-UBT	Abdominal ultrasound
Yan et al. (42)	China	Cross-sectional study	1,185	778	407	43.50 ± 9.94/ 40.90 ± 11.28	–	^13^C-UBT	Abdominal ultrasound
Baeg et al. (43)	South Korea	Cross-sectional study	3,663	–	–	–	–	^13^C-UBT	Hepatic steatosis index (HSI) and NAFLD liver fat score (NAFLD-lfs)
Valadares et al. (44)	Brazil	Cross-sectional study	88	13	72	–	39.1 ± 11/ 39.4 ± 8.1	Rapid Urease Test	liver biopsy
Kang et al. (45)	USA	Cross-sectional study	5,404			46.1 ± 0.15/ 41.5 ± 0.18	48.4 ± 0.26/ 40.2 ± 0.12	Serum Hp specific antibodies	Abdominal ultrasound
Han et al. (46)	South Korea	Cohort	1,784	1,483	301	–	55.9 ± 9.3/ 54.9 ± 10.1	Serum Hp specific antibodies	CAP
Yu et al. (47)	China	Cross-sectional study	20,389	11,969	8,420	50.20 ± 12.13/ 46.45 ± 13.60	–	^13^C-UBT	Abdominal ultrasound
Okushin et al. (48)	Japanese	Cross-sectional study	13,737	7,419	6,318	–	–	Serum Hp specific antibodies	Abdominal ultrasound
Wang et al. (49)	China	Cross-sectional study	1,898	1,217	681	–	37.04 ± 0.289/ 37.28 ± 0.210	^13^C-UBT	Abdominal ultrasound

### 3.2 Study quality

Sixteen out of the 19 cross-sectional studies attained JBI scores of 14 or higher, while the remaining three achieved scores of 10, 13, and 13, respectively, indicating the overall high quality of the cross-sectional studies included. Among the seven cohort studies, two received NOS scores of 3, deemed as low quality; one study scored 4, two scored 5, and one scored 6, categorized as medium quality, while one study with an NOS score of 7 was considered high quality. Additionally, in the eight case-control studies, one study with an NOS score of 5 and two studies with a score of 6 were classified as medium quality. Two studies with an NOS score of 7 and three with a score of 8 were deemed high quality among the remaining case-control studies (Supplementary Tables 1–3).

Sixteen out of the 19 cross-sectional studies were rated as high quality with JBI scores of 14 and above. The remaining three studies obtained JBI scores of 10, 13, and 13, respectively, suggesting a generally high quality of the cross-sectional studies included. Regarding the cohort studies, two out of the seven were assessed as low quality, each receiving NOS scores of 3. One study had a NOS score of 4, two studies had a NOS score of 5, and one study had a NOS score of 6, all of which were evaluated as medium quality. Furthermore, one study with an NOS score of 7 was assessed as high quality. Among the case-control studies, one study with an NOS score of 5 and two studies with an NOS score of 6 were considered of medium quality. Additionally, two studies with a NOS score of 7 and three studies with a NOS score of 8 were evaluated as high quality (Supplementary Tables 1–3).

#### 3.2.1 *H. pylori* infection and occurrence of NAFLD

**FIGURE 2:**
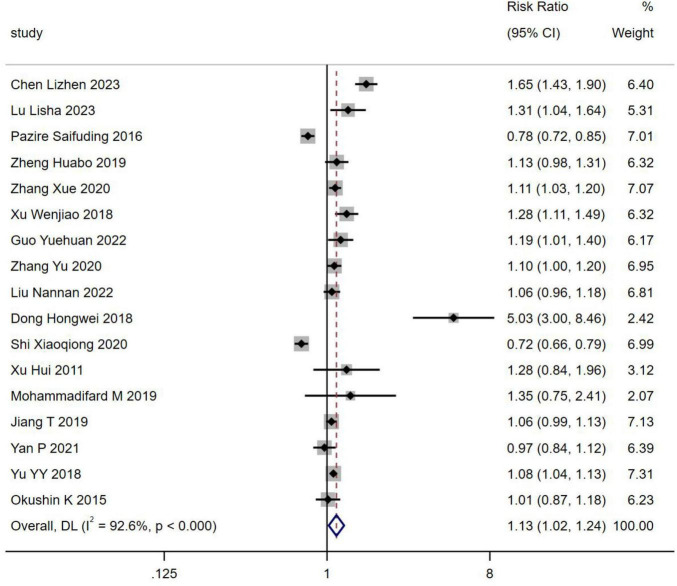
Forest plots for *H. pylori* infection and occurrence of NAFLD.

Seventeen studies, encompassing 74,928 patients, reported the incidence of *H. pylori* infection in individuals with NAFLD. The prevalence of *H. pylori* infection was found to be higher in NAFLD patients compared to those without NAFLD [RR = 1.13, 95% CI (1.02, 1.24), *Z* = 2.395, *P* = 0.017] (Figure 2).

#### 3.2.2 NAFLD and occurrence of *H. pylori* infection

**FIGURE 3:**
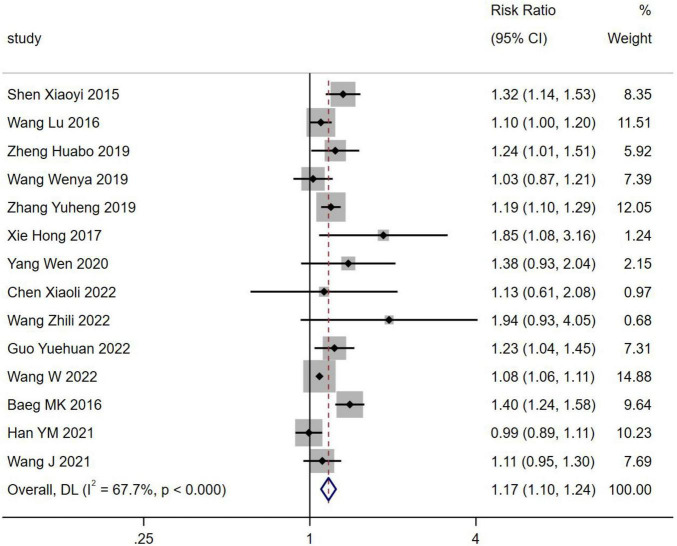
Forest plots for NAFLD and occurrence of *H. pylori* infection.

Fourteen studies reported the incidence of NAFLD in *H. pylori* infection involving 94,950 patients. The incidence of NAFLD was higher in *H. pylori* infection than in *H. pylori* negativity [RR = 1.17, 95% CI (1.10, 1.24), *Z* = 4.897, *P* < 0.001] (Figure 3).

#### 3.2.3 Bias assessment

**FIGURE 4:**
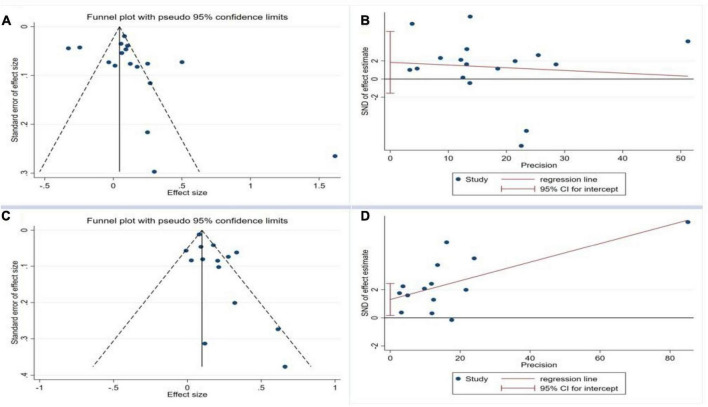
Funnel plot and Egger test for the publication bias test of the included studies. **(A)** Funnel plot for *H. pylori* infection and occurrence of NAFLD; **(B)** Egger test for *H. pylori* infection and occurrence of NAFLD; **(C)** Funnel plot for NAFLD and occurrence of *H. pylori* Infection; **(D)** Egger test for NAFLD and occurrence of *H. pylori* infection.

To assess publication bias, a funnel plot and Egger’s test were employed to scrutinize the incorporation of literature concerning the association between *H. pylori* infection and the onset of NAFLD, as well as the presence of *H. pylori* infection in individuals with NAFLD. The funnel plot exhibited an asymmetrical distribution of data points on both sides of the symmetry axis, suggesting the potential presence of publication bias among the literature included in this analysis (Figures 4A–D). Furthermore, the egger test demonstrated an asymmetrical distribution of scatter points above and below the symmetry axis with the middle line, adding further evidence to the presence of publication bias.

#### 3.2.4 Sensitivity analysis

**FIGURE 5:**
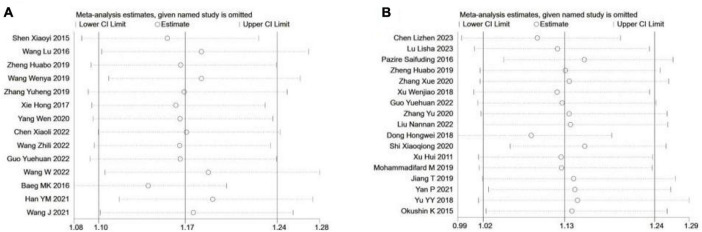
Sensitivity analysis of included studies. **(A)** *H. pylori* Infection and OCCURRENCE of NAFLD; **(B)** NAFLD and occurrence of *H. pylori* infection.

All point estimates for the occurrence of NAFLD in *H. pylori* infections and the occurrence of *H. pylori* positivity in NAFLD fell within the 95% CI of the combined effect sizes, which indicates that the results of the present study are stable (Figures 5A, B).

#### 3.2.5 *H. pylori* Infection and BMI

The BMI was found to be elevated in patients positive for *H. pylori* infection compared to those negative for the infection [WMD = 0.92, 95% CI (0.55, 1.29), *Z* = 4.859, *P* < 0.001] (Supplementary Figure 1).

#### 3.2.6 NAFLD and BMI

BMI was higher in NAFLD patients than in non-NAFLD patients [WMD = 3.05, 95% CI (2.13, 3.97), *Z* = 6.508, *P* < 0.001] (Supplementary Figure 2).

#### 3.2.7 *H. pylori* Infection and FPG

There was no statistically significant variance in FPG levels between patients with positive *H. pylori* infection and those without the infection [WMD = 0.54, 95% CI (−1.13, 2.22), *Z* = 0.635, *P* = 0.525] (Supplementary Figure 3).

#### 3.2.8 NAFLD and FPG

It showed no statistically significant difference in FPG in NAFLD patients compared to non-NAFLD patients [WMD = 1.86, 95% CI (−1.83, 5.56), *Z* = 0.987, *P* = 0.324] (Supplementary Figure 4).

#### 3.2.9 *H. pylori* Infection and liver function (ALT, AST, GGT)

ALT and GGT were higher in *H. pylori* infection-positive patients than in *H. pylori*-negative patients [WMD = 0.60, 95% CI (0.59, 0.61), *Z* = 155.549, *P* < 0.001; WMD = 2.37, 95% CI (1.29, 3.45), *Z* = 4.307, *P* < 0.001]. AST did not differ between the two groups (Supplementary Figure 5).

#### 3.2.10 NAFLD and liver function (ALB, ALT, AST, GGT, TBIL)

ALT, AST and GGT was higher in NAFLD patients than in non-NAFLD patients [WMD = 10.21, 95% CI (6.59, 13.83), *Z* = 5.533, *P* < 0.001; WMD = 3.95, 95% CI (3.11, 4.78), *Z* = 9.311, *P* < 0.001; WMD = 12.88, 95% CI (6.5, 19.26), *Z* = 3.958, *P* < 0.001]. TBIL and ALB did not differ between the two groups [WMD = 0.69, 95% CI (−0.24, 1.62), *Z* = 1.460, *P* = 0.144; WMD = 0.01, 95% CI (−0.04, 0.06), *Z* = 0.480, *P* = 0.631] (Supplementary Figure 6).

#### 3.2.11 *H. pylori* Infection and kidney function (UA, Cr, BUA)

UA, Cr and BUA was higher in *H. pylori* infection-positive patients than in *H. pylori*-negative patients [WMD = 6.19, 95% CI (0.50, 11.87), *Z* = 2.133, *P* = 0.033; WMD = 0.59, 95% CI (0.30, 0.88), *Z* = 3.966, *P* < 0.001; WMD = 0.07, 95% CI (0.05, 0.09), *Z* = 6.649, *P* < 0.001] (Supplementary Figure 7).

#### 3.2.12 NAFLD and kidney function (UA, BUA)

UA of NAFLD patients was higher than that of non-NAFLD patients [WMD = 63.77, 95% CI (47.58, 79.97), *Z* = 7.717, *P* < 0.001]. There was no statistical significance in BUA between the two groups [WMD = 0.14, 95% CI (−0.11, 0.39), *Z* = 1.117, *P* = 0.264] (Supplementary Figure 8).

#### 3.2.13 *H. pylori* Infection and blood lipid (TG, TC, HDL, LDL)

TC, HDL, LDL of *H. pylori* infection-positive patients was higher than that of *H. pylori*-negative patients [WMD = 0.84, 95% CI (0.10, 0.59), *Z* = 2.213, *P* = 0.027; WMD = −0.27 95% CI (−0.49, −0.05), *Z* = −2.427, *P* = 0.015; WMD = 0.11 95% CI (0.06 0.17), *Z* = 3.882, *P* < 0.001] (Supplementary Figure 9). There was no significant difference in the TG between the two groups [WMD = 0.81, 95% CI (−1.59, 3.21), *Z* = 0.661, *P* = 0.508].

#### 3.2.14 NAFLD and blood lipid (TG, TC, HDL, LDL)

TG and LDL were higher in NAFLD patients than in non-NAFLD patients [WMD = 0.94, 95% CI (0.80, 1.08), *Z* = 13.296, *P* < 0.001; WMD = 0.35 95% CI (0.17 0.53), *Z* = 3.793, *P* < 0.001]. There was no significant difference in TC and HDL between the two groups [WMD = 2.22, 95% CI (−0.54, 4.99), *Z* = 1.574, *P* = 0.115; WMD = −1.05 95% CI (−2.25 0.16), *Z* = −1.707, *P* = 0.088] (Supplementary Figure 10).

#### 3.2.15 *H. pylori* and blood pressure

DBP was higher in *H. pylori* infection-positive patients than in *H. pylori*-negative patients [WMD = 1.01, 95% CI (0.11, 1.91), *Z* = 2.211, *P* = 0.027]. SBP did not differ between the two groups[WMD = 1.51, 95% CI (−1.18, 4.19), *Z* = 1.101, *P* = 0.271] (Supplementary Figure 11).

#### 3.2.16 NAFLD and blood pressure

SBP and DBP were higher in NAFLD patients than in non-NAFLD patients [WMD = 8.03, 95% CI (6.50, 9.55), *Z* = 10.298, *P* < 0.001; WMD = 5.71, 95% CI (4.02, 7.39), *Z* = 6.645, *P* < 0.001] (Supplementary Figure 12).

## 4 Discussion

Our comprehensive meta-analysis has identified *H. pylori* as a significant risk factor for individuals prone to NAFLD. Additionally, the prevalence of *H. pylori* infection among NAFLD patients was determined to be 1.13 times higher compared to those without NAFLD. Through a bidirectional meta-analysis of the latest published studies, employing a meticulous search strategy and stringent selection criteria, we have amassed substantial evidence supporting the correlation between *H. pylori* infection and NAFLD. Notably, our meta-analysis boasts a larger sample size compared to previous investigations, enhancing the robustness and currency of our findings.

MetS, comprising overweight/obesity, type 2 diabetes mellitus (T2DM), and metabolic dysregulation, plays a pivotal role in the onset of NAFLD (50, 51). Moreover, a strong correlation exists between MetS and *H. pylori* infection (52). Through our meta-analysis, we have established a relationship between *H. pylori* infection and NAFLD. This enhances our comprehension of the underlying mechanisms linking *H. pylori* infection, MetS, and NAFLD, an aspect that has not been extensively explored in prior meta-analyses.

There is a lack of direct experimental mechanistic evidence to support the effect of *H. pylori* infection on NAFLD. Disruption of the gastrointestinal epithelium and transport of *H. pylori*-associated metabolites through the portal flow to the liver activate the toll-like receptor inflammatory process that may develop NAFLD (53). In particular, low-grade chronic inflammation in the gastric mucosa may exacerbate and promote the local and systemic release of several pro-inflammatory cytokines, thereby exacerbating systemic insulin resistance (IR), increasing disorders of lipid metabolism (adipocytokines and lipid metabolism), increasing intestinal permeability, and altering the composition of the gut microbiome. Inflammatory cytokines are key in the pathogenesis of both *H. pylori* infection and NAFLD (54, 55). Persistent *H. pylori* infection may lead to chronic low-level inflammation and increased expression of NOD-like receptor protein 3 (NLRP3) inflammatory vesicles, as well as inflammatory cytokines such as interleukin-1β (IL-1β), IL-6 and TNF-α (54, 55). However, the exact relationship between *H. pylori* infection and serum adipocytokines remains uncertain, and further extensive prospective studies are needed to establish a conclusive link. IR is a key factor in the development of NAFLD, contributing significantly to hepatic triglyceride accumulation, inflammatory cascade response and progression of liver fibrosis (56). Meta-analyses suggest a possible correlation between *H. pylori* infection and IR (57). Hepatocellular steatosis, characterized by disturbances in hepatocellular lipid metabolism, is the main pathological manifestation of NAFLD (9). A comprehensive analysis using a large cohort propensity score-matched study suggested that eradication of *H. pylori* may mitigate the deterioration of lipid metabolism. However, lipid levels did not fully recover to those observed in uninfected individuals (58). There is strong evidence that *H. pylori* infection affects the integrity of the intestinal barrier. In an experiment involving mice fed a high-fat diet and infected with *H. pylori*, a significant reduction in the expression of tight junction proteins in the intestinal barrier was observed. This reduction was attributed to an increase in CagA-containing exosomes, leading to increased intestinal permeability (59). *H. pylori* infection may alter the composition of the intestinal microbiota by altering the anaplasmosis, Lactobacillus, Aspergillus, Rickettsia and Actinomycetes groups, as observed in obese patients (60).

Our meta-analysis has several limitations. Firstly, the majority of the studies included only presented cross-sectional data, which could introduce recall and selection biases. As a result, the findings can only suggest a potential association between *H. pylori* infection and NAFLD. Secondly, only a subset of the studies accounted for confounding factors in multivariate regression analyses. This lack of adjustment may introduce confounding variables and affect the accuracy of the results. Thirdly, the presence of significant heterogeneity across the studies could potentially undermine the reliability of the pooled odds ratio estimates. Fourthly, there are disparities in the diagnostic methods for *H. pylori* infection and NAFLD among the included studies. Fifthly, although we included all available studies in our meta-analysis, the number of studies and participants may still be insufficient. Therefore, it is essential to interpret the results of this meta-analysis critically and cautiously, and further multicenter prospective studies are necessary to validate the main findings.

Despite these limitations, our meta-analysis also has important strengths. We implemented a rigorous search strategy and strict inclusion criteria, including all available evidence published to date. To the best of our knowledge, our meta-analysis is the largest and most recent updated meta-analysis to date designed to investigate the association between *H. pylori* infection and NAFLD risk. Second, we used standardized risk estimates from all included studies to achieve a consistent combination of estimates between studies. In addition, our study was registered in advance on the PROSPERO platform, and most of the included studies were of high quality, indicating that our results are reliable.

Besides, we also proved the relationship between NAFLD and metabolic disorders such as BMI, ALT, AST, GGT, UA, TG, LDL, DBP and SBP by meta-analysis. MetS has been shown to be the strongest risk factor for NAFLD and NASH (61). In 2020, the term NAFLD was replaced with metabolism-associated fatty liver disease (MAFLD), a change that garnered widespread recognition within the academic community globally (62–65).

In summary, there is clear evidence of a substantial and bidirectional relationship between *H. pylori* infection and the susceptibility to NAFLD. This underscores the importance for clinicians to pay close attention to this correlation. However, additional research is needed to bolster and clarify this association, as well as to elucidate the underlying mechanisms.

## Data availability statement

The original contributions presented in this study are included in the article/Supplementary material, further inquiries can be directed to the corresponding author.

## Author contributions

DZ: Conceptualization, Data curation, Formal analysis, Investigation, Methodology, Project administration, Resources, Software, Supervision, Validation, Visualization, Writing – original draft, Writing – review & editing. QW: Methodology, Project administration, Resources, Software, Supervision, Validation, Visualization, Writing – review & editing. FB: Conceptualization, Data curation, Formal analysis, Funding acquisition, Investigation, Methodology, Project administration, Resources, Software, Supervision, Validation, Visualization, Writing – original draft, Writing – review & editing.

## Registration and protocol

This study was registered on the PROSPERO with a registration number CRD42023488399.

## References

[1] Crowe S. *Helicobacter pylori* infection. *N Engl J Med.* (2019) 380:1158–65. 30893536 10.1056/NEJMcp1710945

[2] Usui Y Taniyama Y Endo M. *Helicobacter pylori*, homologous-recombination genes, and gastric cancer. *N Engl J Med.* (2023) 388:1181–90. 36988593 10.1056/NEJMoa2211807

[3] Zhou X Lyu N Zhu H Cai Q Kong X Xie P Large-scale, national, family-based epidemiological study on *Helicobacter pylori* infection in China: The time to change practice for related disease prevention. *Gut.* (2023) 72:855–69. 10.1136/gutjnl-2022-328965 36690433 PMC10086479

[4] Li Y Choi H Leung K Jiang F Graham D Leung W. Global prevalence of *Helicobacter pylori* infection between 1980 and 2022: A systematic review and meta-analysis. *Lancet Gastroenterol Hepatol.* (2023) 8:553–64. 10.1016/S2468-1253(23)00070-5 37086739

[5] Helicobacter pylori Group of the Society of Gastroenterology of the Chinese Medical Association. Sixth national consensus report on the management of *Helicobacter pylori* infection (non-eradication section). *Chin J Gastroenterol.* (2022) 42:289–303.

[6] Younossi Z. Non-alcoholic fatty liver disease –A global public health perspective. *J Hepatol.* (2018) 70:531–44. 30414863 10.1016/j.jhep.2018.10.033

[7] Riazi K Azhari H Charette J Underwood F King J Afshar E The prevalence and incidence of NAFLD worldwide: A systematic review and meta-analysis. *Lancet Gastroenterol Hepatol.* (2022) 7:851–61. 35798021 10.1016/S2468-1253(22)00165-0

[8] Huang D El-Serag H Loomba R. Global epidemiology of NAFLD related HCC: Trends, predictions, risk factors and prevention. *Nat Rev Gastroenterol Hepatol.* (2021) 18:223–38. 10.1038/s41575-020-00381-6 33349658 PMC8016738

[9] Powell E Wong V Rinella M. Non-alcoholic fatty liver disease. *Lancet.* (2021) 397:2212–24. 33894145 10.1016/S0140-6736(20)32511-3

[10] Caussy C Aubin A Loomba R. The relationship between type 2 diabetes, NAFLD, and cardiovascular risk. *Curr Diab Rep.* (2021) 21:15. 10.1007/s11892-021-01383-7 PMC8805985 33742318

[11] Polyzos S Kountouras J Papatheodorou A. *Helicobacter pylori* infection in patients with nonalcoholic fatty liver disease. *Metabolism.* (2013) 62:121–6. 10.1016/j.metabol.2012.06.007 22841522

[12] Fan N Peng L Xia Z Zhang L Wang Y Peng Y. *Helicobacter pylori* infection is not associated with non-alcoholic fatty liver disease: A cross-sectional study in China. *Front Microbiol.* (2018) 9:73. 10.3389/fmicb.2018.00073 29445363 PMC5797778

[13] Wernly S Wernly B Semmler G Völkerer A Rezar R Semmler L Nonalcoholic fatty liver disease is not independently associated with *Helicobacter pylori* in a central European screening cohort. *Minerva Med.* (2022) 113:936–49. 10.23736/s0026-4806.22.07928-9 35384436

[14] Kim T Sinn D Min Y Son H Kim J Chang Y A cohort study on *Helicobacter pylori* infection associated with nonalcoholic fatty liver disease. *J Gastroenterol.* (2017) 52:1201–10. 10.1007/s00535-017-1337-y 28382402

[15] Polyzos S Nikolopoulos P Stogianni A Romiopoulos I Katsinelos P Kountouras J. Effect of *Helicobacter pylori* eradication on hepatic steatosis, NAFLD fibrosis score and HSENSI in patients with nonalcoholic steatohepatitis: A MR imaging-based pilot open-label study. *Arq Gastroenterol.* (2014) 51:261–8. 10.1590/s0004-28032014000300017 25296089

[16] Chen L Cai Y Zhang J. Analysis of the correlation between nonalcoholic fatty liver disease and *Helicobacter pylori* infection. *Chin J Pathog Biol.* (2023) 18:1434–7.

[17] Lisa L Zhang Z Wang G. Study on the correlation between *Helicobacter pylori* infection and nonalcoholic fatty liver disease in an enterprise population in Lanzhou City. *J Med Forum.* (2023) 44:61–6.

[18] Pazizhe S. *Correlation analysis of non-alcoholic fatty liver disease and Helicobacter pylori* infection. Ürümqi: Xinjiang Medical University (2016).

[19] Shen X. *Study on the correlation between non-alcoholic fatty liver disease and Helicobacter pylori* infection. Suzhou: Suzhou University (2015).

[20] Wang L. *Investigation on the correlation between Helicobacter pylori* infection and non-alcoholic fatty liver disease in the physical examination population in Taiyuan City. Jinzhong: Shanxi Medical University (2016).

[21] Zheng H. *Cohort study on the correlation between Helicobacter pylori* infection and non-alcoholic fatty liver disease. Wuhan: Huazhong University of Science and Technology (2019).

[22] Zhang X. *Analysis of the correlation between Helicobacter pylori* infection and non-alcoholic fatty liver disease. Lanzhou: Northwest University for Nationalities (2020).

[23] Wang W. Analysis of the correlation between *Helicobacter pylori* infection and nonalcoholic fatty liver disease. *Modern Med.* (2019) 47:1494–7.

[24] Xu W Li C. Correlation analysis of the correlation between *Helicobacter pylori* infection and nonalcoholic fatty liver disease. *Family Med.* (2018) 58:73–4.

[25] Peng C Sheng X Ding H. Study on the correlation between *Helicobacter pylori* infection and nonalcoholic fatty liver disease. *J Med Res.* (2014) 43:153–6.

[26] Wu C Chen X Wu J. Study on the correlation between *Helicobacter pylori* infection and non-alcoholic fatty liver disease. *World Digest Recent Med Inf.* (2018) 18:155.

[27] Zhang Y Ding S. Relationship between *Helicobacter pylori* and nonalcoholic fatty liver disease. *J Med Forum.* (2019) 40:34–6.

[28] Xie H Ye Y. Study on the correlation between *Helicobacter pylori* and nonalcoholic fatty liver disease. *Chin For Med Res.* (2017) 15:13–4.

[29] Yang W Wang Y Zhang Z. Study on *Helicobacter pylori* infection associated with nonalcoholic fatty liver disease. *Chin J Health Care Med.* (2020) 22:212–3.

[30] Chen X Chen T Li H. Correlation analysis of *Helicobacter pylori* infection with non-alcoholic fatty liver disease and lipid index. *J Med Forum.* (2022) 43:1–4.

[31] Wang Z Lu S Yang L. Analysis of the correlation between *Helicobacter pylori* infection and non-alcoholic fatty liver disease and lipid metabolism. *J Dalian Med Univers.* (2022) 44:48–51+57.

[32] Guo Y. *Study on the correlation between Helicobacter pylori* infection and non-alcoholic fatty liver disease. Hefei: Anhui Medical University (2022).

[33] Zhang Y Xu R Li C. Analysis of risk factors of non-alcoholic fatty liver disease and its correlation with *Helicobacter pylori* in Shenzhen medical examination population. *Gansu Med.* (2020) 39:691–4.

[34] Liu N. *Study on the correlation between Helicobacter pylori* infection and non-alcoholic fatty liver disease in Handan area. Hebei: Hebei North College (2022).

[35] Liu A Wang L Zhang Y. Correlation between non-alcoholic fatty liver disease and *Helicobacter pylori* infection. *J Gastroenterol Hepatol.* (2014) 23:1451–4.

[36] Dong H. Analysis of the correlation between non-alcoholic fatty liver disease and *Helicobacter pylori* infection. *World Digest Recent Med Inf.* (2018) 18:21–2.

[37] Shi X Wu J Wei M. Correlation between nonalcoholic fatty liver disease and *Helicobacter pylori* (Hp) infection in Science City area. *World Digest Recent Med Inf.* (2020) 20:119.

[38] Xu F. Relationship between *Helicobacter pylori* infection and nonalcoholic fatty liver disease. *China Aesth Med.* (2011) 20:69–70.

[39] Mohammadifard M Saremi Z Rastgoo M Akbari E. Relevance between *Helicobacter pylori* infection and non-alcoholic fatty liver disease in Birjand, Iran. *J Med Life.* (2019) 12:168–72. 10.25122/jml-2019-0012 31406519 PMC6685302

[40] Jiang T Chen X Xia C Liu H Yan H Wang G Association between *Helicobacter pylori* infection and non-alcoholic fatty liver disease in North Chinese: A cross-sectional study. *Sci Rep.* (2019) 9:4874. 10.1038/s41598-019-41371-2 30890750 PMC6425019

[41] Wang W Fan M Gong R Zhang Y Zeng J Xu S *Helicobacter pylori* infection is not an independent risk factor of non-alcoholic fatty liver disease in China. *BMC Gastroenterol.* (2022) 22:81. 10.1186/s12876-022-02148-6 35209867 PMC8867781

[42] Yan P Yu B Li M Zhao W. Association between nonalcoholic fatty liver disease and *Helicobacter pylori* infection in Dali city, China. *Saudi Med J.* (2021) 42:735–41. 10.15537/smj.2021.42.7.20210040 34187917 PMC9195526

[43] Baeg M Yoon S Ko S Noh Y Lee I Choi M. *Helicobacter pylori* infection is not associated with nonalcoholic fatty liver disease. *World J Gastroenterol.* (2016) 22:2592–600. 10.3748/wjg.v22.i8.2592 26937147 PMC4768205

[44] Valadares E Gestic M Utrini M Chaim F Chaim E Cazzo E. Is *Helicobacter pylori* infection associated with non-alcoholic fatty liver disease in individuals undergoing bariatric surgery? Cross-sectional study. *Sao Paulo Med J.* (2023) 141:e2022517. 10.1590/1516-3180.2022.0517.R1.14122022 37042863 PMC10085533

[45] Kang S Kim H Kim D Ahmed A. Association between cagA negative *Helicobacter pylori* status and nonalcoholic fatty liver disease among adults in the United States. *PLoS One.* (2018) 13:e0202325. 10.1371/journal.pone.0202325 30110395 PMC6093702

[46] Han Y Lee J Choi J Kwak M Yang J Chung S The association between *Helicobacter pylori* with nonalcoholic fatty liver disease assessed by controlled attenuation parameter and other metabolic factors. *PLoS One.* (2021) 16:e0260994. 10.1371/journal.pone.0260994 34898613 PMC8668115

[47] Yu Y Cai J Song Z Tong Y Wang J. The associations among *Helicobacter pylori* infection, white blood cell count and nonalcoholic fatty liver disease in a large Chinese population. *Medicine (Baltimore).* (2018) 97:e13271. 10.1097/MD.0000000000013271 30431613 PMC6257485

[48] Okushin K Takahashi Y Yamamichi N Shimamoto T Enooku K Fujinaga H *Helicobacter pylori* infection is not associated with fatty liver disease including non-alcoholic fatty liver disease: A large-scale cross-sectional study in Japan. *BMC Gastroenterol.* (2015) 15:25. 10.1186/s12876-015-0247-9 25880912 PMC4349671

[49] Wang J Dong F Su H Zhu L Shao S Wu J H. pylori is related to NAFLD but only in female: A cross-sectional study. *Int J Med Sci.* (2021) 18:2303–11. 10.7150/ijms.50748 33967606 PMC8100637

[50] Lim S Kim J Targher G. Links between metabolic syndrome and metabolic dysfunction-associated fatty liver disease. *Trends Endocrinol Metab.* (2021) 32:500–14. 33975804 10.1016/j.tem.2021.04.008

[51] Wang X Malhi H. Nonalcoholic fatty liver disease. *Ann Intern Med.* (2018) 169:ITC65–80. 30398639 10.7326/AITC201811060

[52] Franceschi F Gasbarrini A Polyzos S Kountouras J. Extragastric diseases and *Helicobacter pylori*. *Helicobacter*. (2015) 20:40–6. 26372824 10.1111/hel.12256

[53] Doulberis M Srivastava S Polyzos S Kountouras J Papaefthymiou A Klukowska-Rötzler J Active *Helicobacter pylori* infection is independently associated with nonalcoholic steatohepatitis in morbidly obese patients. *J Clin Med.* (2020) 9:933. 10.3390/jcm9040933 32235601 PMC7230908

[54] Pérez-Figueroa E Torres J Sánchez-Zauco N. Activation of NLRP3 inflammasome in human neutrophils by *Helicobacter pylori* infection. *Innate Immun.* (2016) 22:103–12. 10.1177/1753425915619475 26610398

[55] Li H Liu N Li J Wang M Tan J Dong B Bicyclol ameliorates advanced liver diseases in murine models via inhibiting the IL-6/STAT3 signaling pathway. *Biomed Pharmacother.* (2022) 150:113083. 10.1016/j.biopha.2022.113083 35658240

[56] Watt M Miotto P De Nardo W Montgomery M. The liver as an endocrine organ-linking NAFLD and insulin resistance. *Endocr Rev.* (2019) 40:1367–93. 10.1210/er.2019-00034 31098621

[57] Azami M Baradaran H Dehghanbanadaki H Kohnepoushi P Saed L Moradkhani A Association of *Helicobacter pylori* infection with the risk of metabolic syndrome and insulin resistance: An updated systematic review and meta-analysis. *Diabetol Metab Syndr.* (2021) 13:145. 10.1186/s13098-021-00765-x 34922625 PMC8684139

[58] Watanabe J Hamasaki M Kotani K. The effect of *Helicobacter pylori* eradication on lipid levels: A meta-analysis. *J Clin Med.* (2021) 10:904. 10.3390/jcm10050904 33668848 PMC7956592

[59] Guo Y Xu C Gong R Hu T Zhang X Xie X Exosomal CagA from *Helicobacter pylori* aggravates intestinal epithelium barrier dysfunction in chronic colitis by facilitating Claudin-2 expression. *Gut Pathog.* (2022) 14:13. 10.1186/s13099-022-00486-0 35331316 PMC8944046

[60] Mavilia-Scranton M Wu G Dharan M. Impact of *Helicobacter pylori* infection on the pathogenesis and management of nonalcoholic fatty liver disease. *J Clin Transl Hepatol.* (2023) 11:670–4. 10.14218/JCTH.2022.00362 36969902 PMC10037521

[61] Friedman S Neuschwander-Tetri B Rinella M Sanyal A. Mechanisms of NAFLD development and therapeutic strategies. *Nat Med.* (2018) 24:908–22. 29967350 10.1038/s41591-018-0104-9 PMC6553468

[62] Eslam M Sanyal A George J. MAFLD: A consensus-driven proposed nomenclature for metabolic associated fatty liver disease. *Gastroenterology.* (2020) 158:1999–2014.e1. 10.1053/j.gastro.2019.11.312 32044314

[63] Eslam M Newsome P Sarin S Anstee Q Targher G Romero-Gomez M A new definition for metabolic dysfunction-associated fatty liver disease: An international expert consensus statement. *J Hepatol.* (2020) 73:202–9. 32278004 10.1016/j.jhep.2020.03.039

[64] Nan Y An J Bao J Chen H Chen Y Ding H The Chinese society of hepatology position statement on the redefinition of fatty liver disease. *J Hepatol.* (2021) 75:454–61. 34019941 10.1016/j.jhep.2021.05.003

[65] Méndez-Sánchez N Bugianesi E Gish R Lammert F Tilg H Nguyen M Global multi-stakeholder consensus on the redefinition of fatty liver disease. Global multi-stakeholder endorsement of the MAFLD definition. *Lancet Gastroenterol Hepatol.* (2022) 7:388–90. 10.1016/S2468-1253(22)00062-0 35248211

