# Supplementary material for: Bidirectional relationship between Helicobacter pylori infection and nonalcoholic fatty liver disease: insights from a comprehensive meta-analysis
Source: Front Nutr. 2024 Aug 5;11:1410543. doi: 10.3389/fnut.2024.1410543 (PMC11332609; doi:10.3389/fnut.2024.1410543)
Supplement: Supplementary file 1 [file Data_Sheet_1.docx]

Supplementary table 1 Cross-section study quality evaluation

| Cross-section study | Purpose | population | Characteristics of the sample | Inclusion and exclusion criteria | Credibility and validity of the data | Authenticity of the data | Consideration of ethics | Correctness of the statistical methods | Accuracy of the findings | Elaboration of the value of the study | JBI scores |
| --- | --- | --- | --- | --- | --- | --- | --- | --- | --- | --- | --- |
| Chen LZ 2023 | 2 | 1 | 2 | 2 | 1 | 1 | 0 | 2 | 2 | 2 | 15 |
| Shen XY 2015 | 2 | 1 | 2 | 2 | 1 | 1 | 0 | 2 | 1 | 2 | 14 |
| Wang L 2016 | 2 | 1 | 2 | 1 | 1 | 0 | 0 | 2 | 2 | 1 | 13 |
| Zhang X 2020 | 2 | 1 | 2 | 2 | 1 | 0 | 0 | 2 | 2 | 2 | 14 |
| Zhang YH 2019 | 2 | 1 | 1 | 2 | 1 | 1 | 0 | 2 | 2 | 2 | 14 |
| Guo YH 2022 | 2 | 1 | 1 | 2 | 1 | 1 | 2 | 2 | 2 | 2 | 16 |
| Zhang Y 2020 | 2 | 1 | 2 | 2 | 2 | 1 | 0 | 2 | 2 | 1 | 15 |
| Liu NN 2022 | 2 | 1 | 2 | 2 | 0 | 1 | 0 | 2 | 2 | 2 | 14 |
| Liu AN 2014 | 2 | 1 | 2 | 2 | 1 | 0 | 0 | 2 | 2 | 1 | 13 |
| Shi XQ 2020 | 2 | 1 | 1 | 1 | 0 | 0 | 0 | 2 | 2 | 1 | 10 |
| Jiang T 2019 | 2 | 1 | 2 | 0 | 2 | 2 | 2 | 2 | 2 | 2 | 15 |
| Wang W 2022 | 2 | 1 | 2 | 2 | 2 | 1 | 2 | 2 | 2 | 1 | 17 |
| Yan P 2021 | 2 | 1 | 2 | 2 | 2 | 2 | 2 | 2 | 2 | 1 | 18 |
| Baeg MK 2016 | 2 | 1 | 2 | 2 | 1 | 1 | 2 | 2 | 2 | 2 | 17 |
| Valadares EC 2022 | 2 | 1 | 2 | 2 | 0 | 0 | 2 | 2 | 2 | 1 | 14 |
| Kang SJ 2018 | 2 | 2 | 2 | 2 | 1 | 2 | 2 | 2 | 2 | 2 | 19 |
| Yu YY 2018 | 2 | 1 | 1 | 1 | 0 | 2 | 2 | 2 | 2 | 2 | 15 |
| Okushin K 2015 | 2 | 1 | 2 | 1 | 1 | 1 | 2 | 2 | 2 | 1 | 15 |
| Wang J 2021 | 2 | 1 | 2 | 1 | 0 | 2 | 2 | 2 | 2 | 2 | 16 |

Supplementary table 2 cohort study quality evaluation

| study | Selective (0-4 points) | | | | Comparative (0-2 points) | Conclusion (0-3 points) | | | total points |
| --- | --- | --- | --- | --- | --- | --- | --- | --- | --- |
|  | Selection of exposed population | Selection of non-exposed population | Measurement of exposure factors | Whether outcome of interest occurred before intervention | Comparability of exposed and non-exposed groups | Accuracy and unbiased assessment of outcome | Whether the follow-up period is long enough | Adequacy of follow-up |  |
| Zheng HB 2019 | 1 | 1 | 1 | 1 | 1 | 1 | 0 | 1 | 7 |
| Wang WY 2019 | 1 | 1 | 1 | 0 | 1 | 1 | 0 | 0 | 5 |
| Xie H 2017 | 1 | 1 | 1 | 0 | 0 | 0 | 0 | 0 | 3 |
| Yang W 2020 | 1 | 1 | 1 | 0 | 1 | 1 | 0 | 0 | 5 |
| Chen XL 2022 | 1 | 1 | 1 | 0 | 0 | 0 | 0 | 0 | 3 |
| Wang ZL 2022 | 1 | 1 | 1 | 0 | 0 | 1 | 0 | 0 | 4 |
| Han YM 2021 | 1 | 1 | 1 | 0 | 2 | 1 | 0 | 0 | 6 |

Supplementary table 3 case-control quality evaluation

| Study | Selective (0-4 points) | | | | Comparative (0-2 points) | Conclusion (0-3 points) | | | total points |
| --- | --- | --- | --- | --- | --- | --- | --- | --- | --- |
|  | Appropriateness of case identification | Representativeness of cases | Selection of controls | Determination of controls | Comparability of cases and controls | Determination of exposure factors | Method of determining exposure factors | Non-response rate |  |
| Lu LS 2023 | 1 | 1 | 1 | 1 | 1 | 1 | 1 | 1 | 8 |
| Pazizge Saifuddin 2016 | 1 | 1 | 1 | 1 | 1 | 1 | 1 | 1 | 8 |
| Xu WJ 2018 | 0 | 1 | 1 | 1 | 0 | 1 | 1 | 1 | 6 |
| Peng ZY 2014 | 1 | 1 | 1 | 1 | 1 | 1 | 1 | 1 | 8 |
| Wu CJ 2018 | 1 | 1 | 0 | 0 | 0 | 1 | 1 | 1 | 5 |
| Dong HW 2018 | 1 | 1 | 0 | 0 | 1 | 1 | 1 | 1 | 6 |
| Xu H 2011 | 1 | 1 | 1 | 1 | 0 | 1 | 1 | 1 | 7 |
| Mohammadifard M 2019 | 1 | 1 | 1 | 1 | 0 | 1 | 1 | 1 | 7 |

**
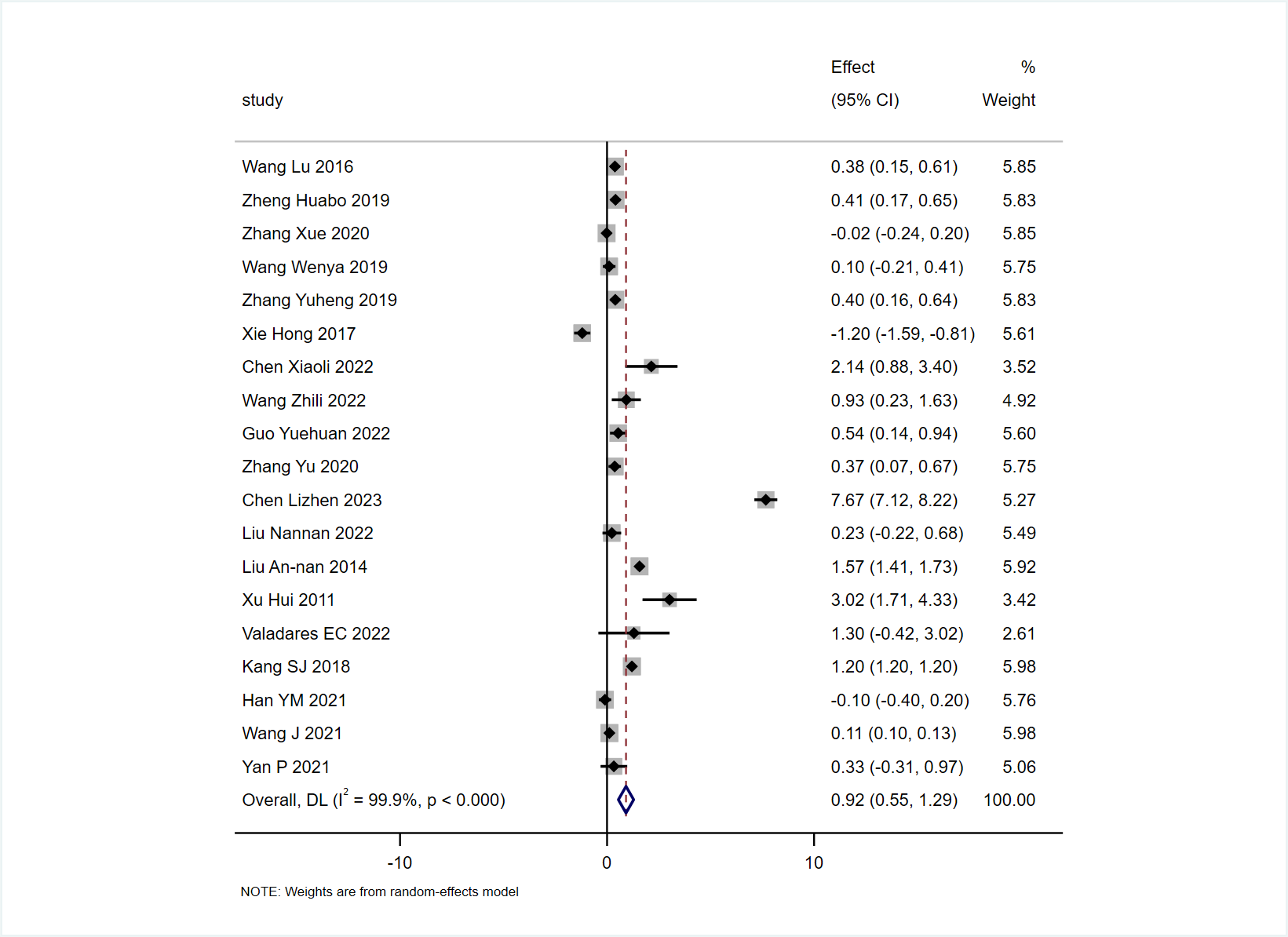
**

Supplementary Figure 1 Forest plots for H. pylori infection and BMI


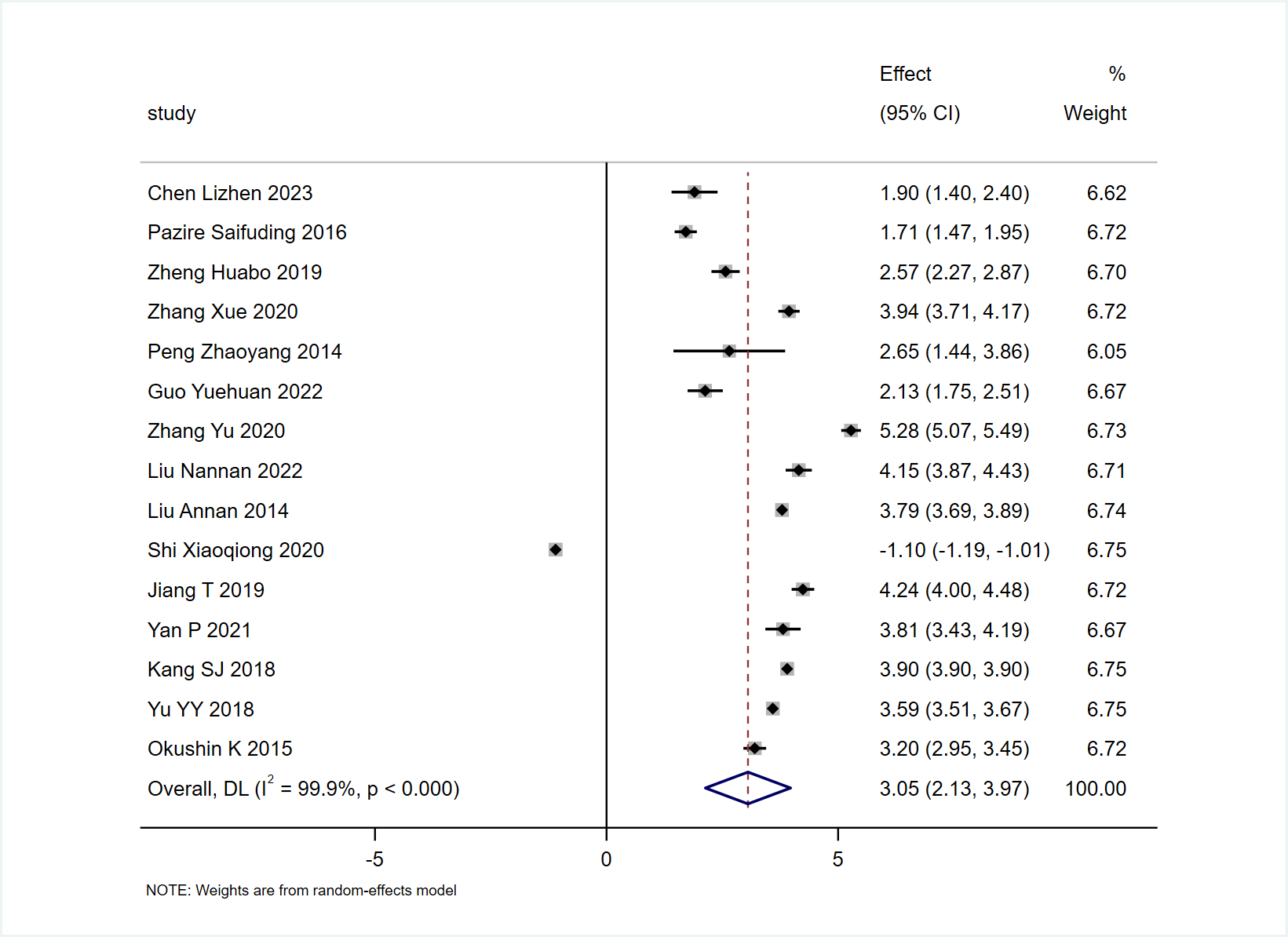


Supplementary Figure 2 Forest plots for NAFLD and BMI

**
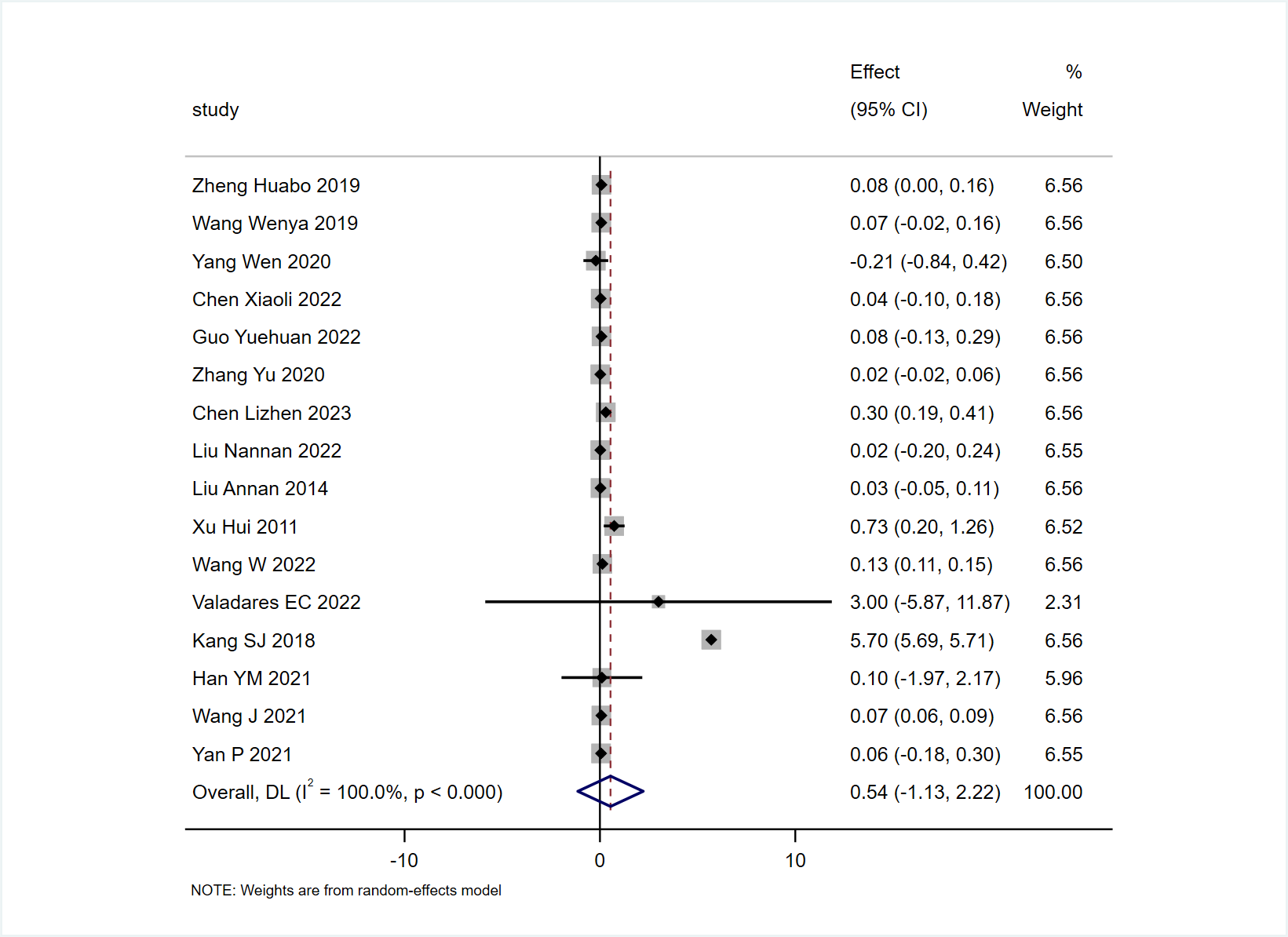
**

Supplementary Figure 3 Forest plots for H. pylori infection and FPG

**
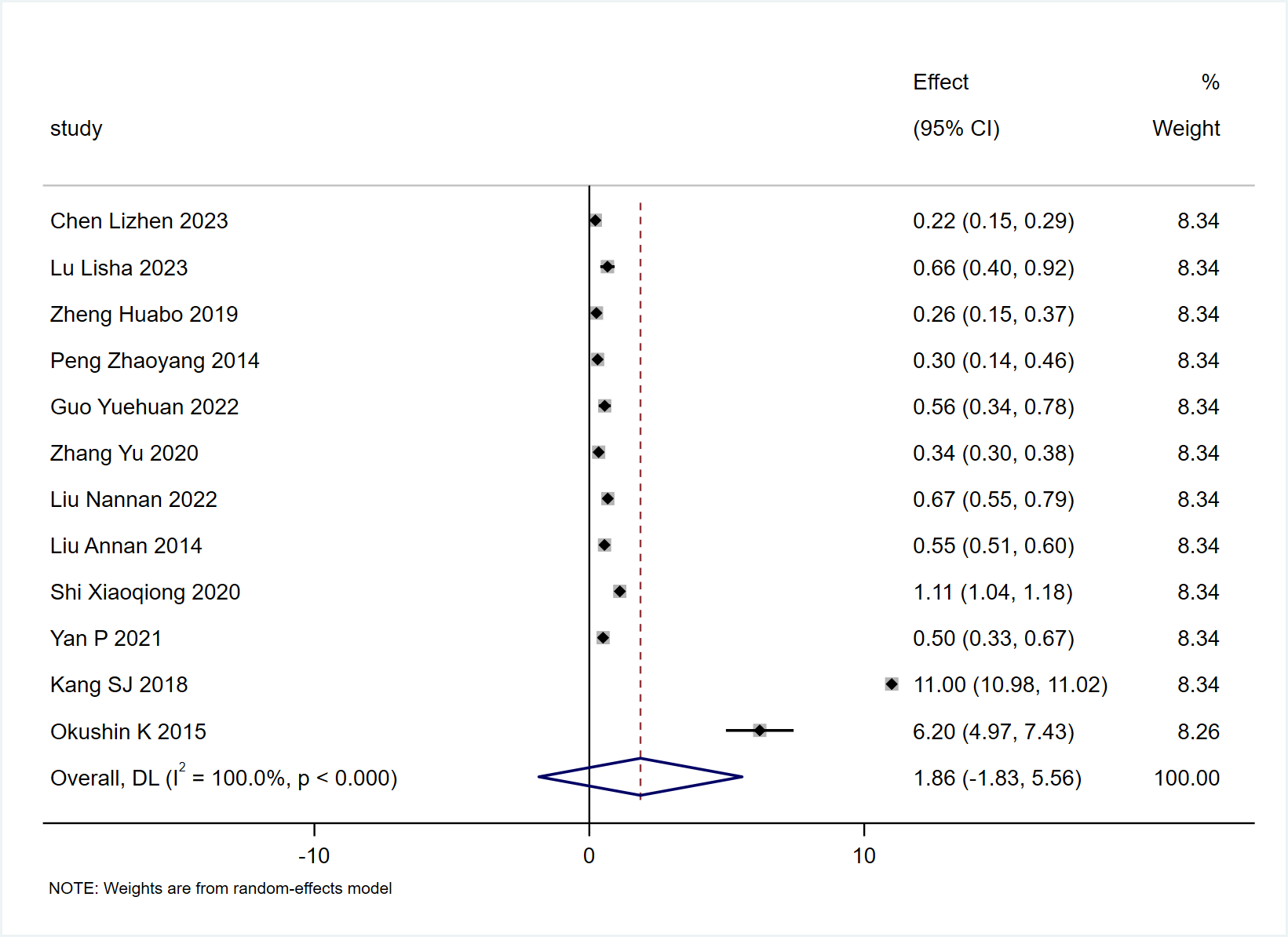
**

Supplementary Figure 4 Forest plots for NAFLD and FPG

**
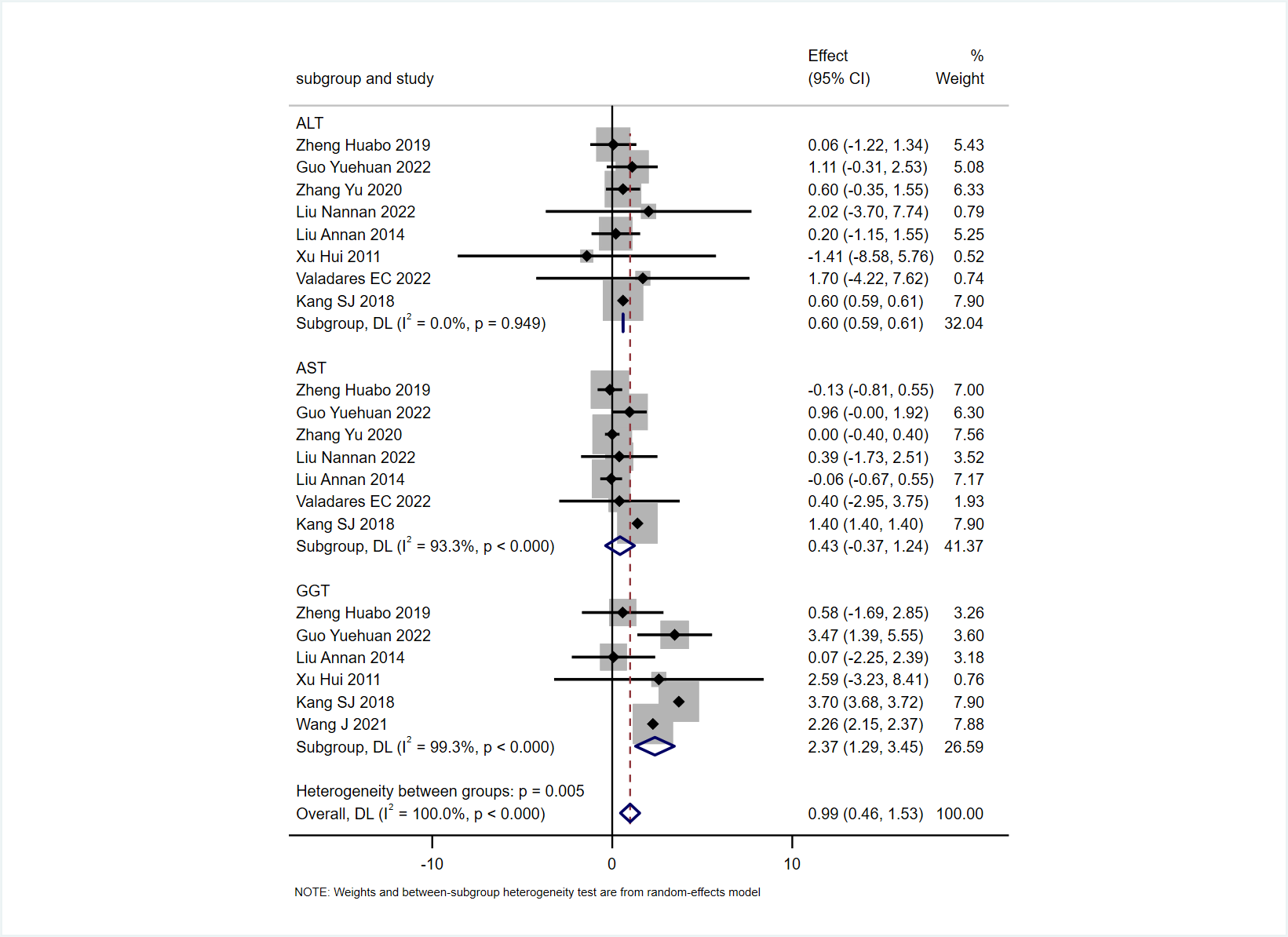
**

Supplementary Figure 5 Forest plots for H. pylori Infection and liver function（ALT, AST, GGT）

**
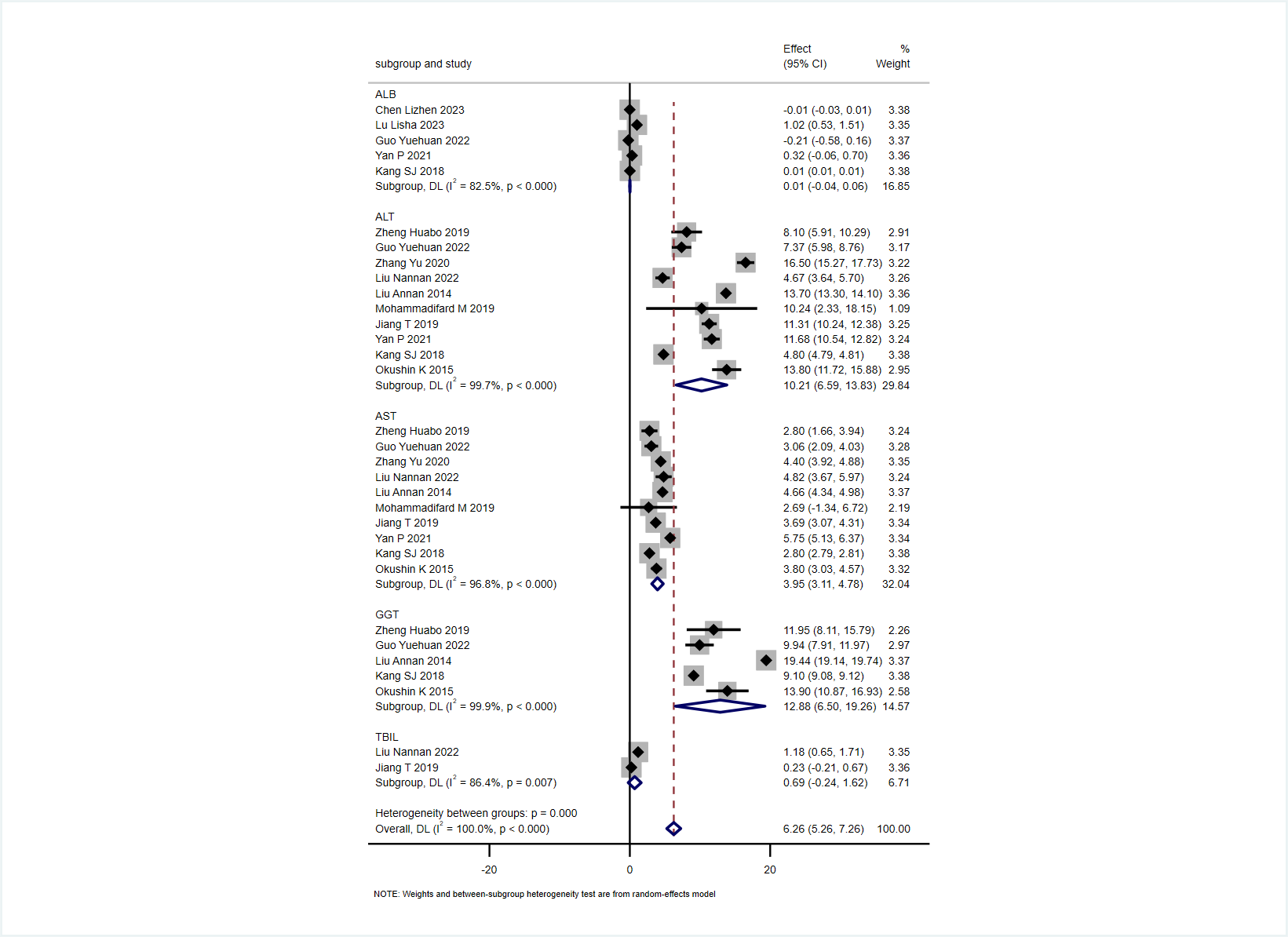
**

Supplementary Figure 6 Forest plots for NAFLD and liver function（ALB, ALT, AST, GGT, TBIL）

**
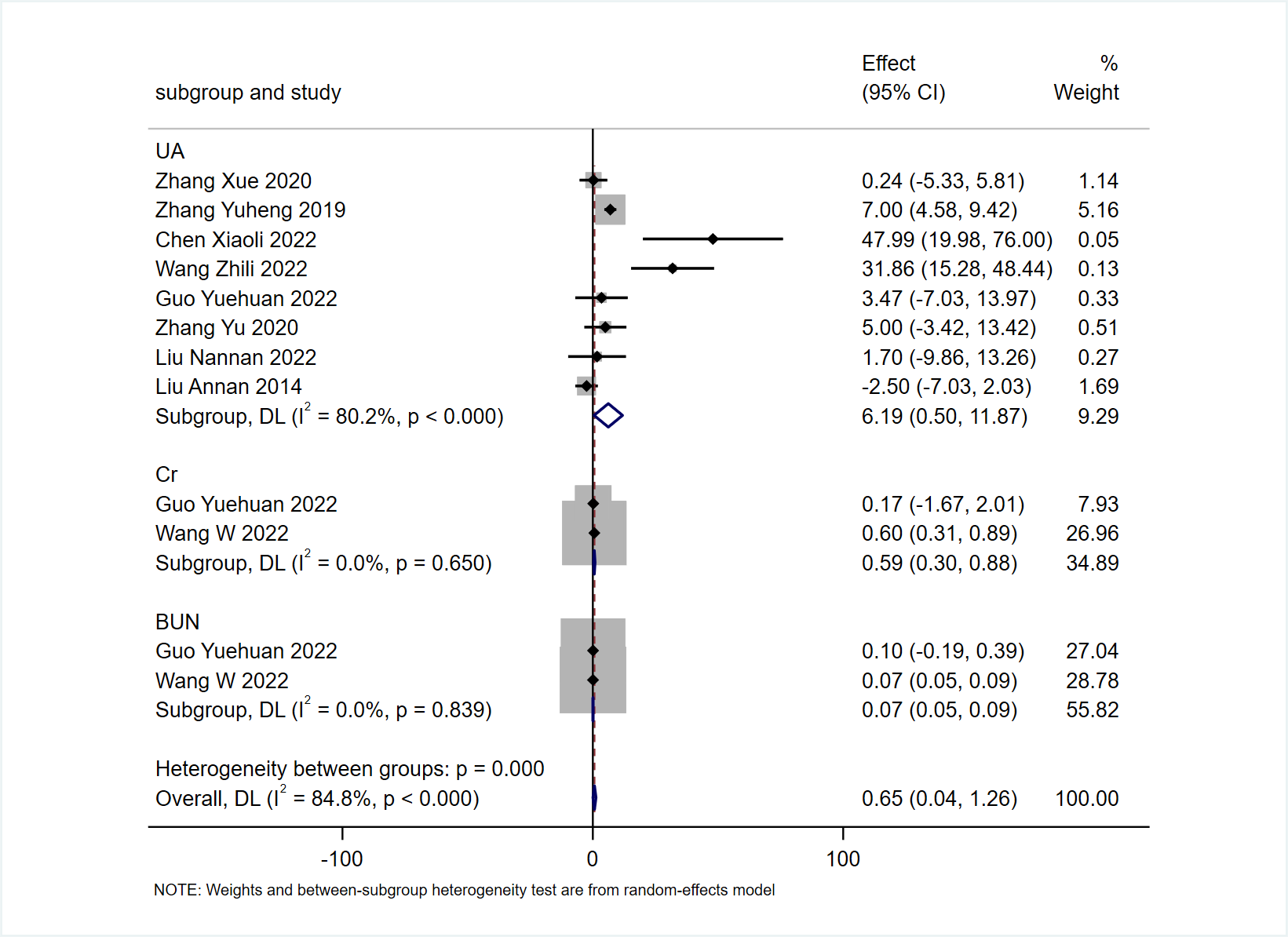
**

Supplementary Figure 7 Forest plots for H. pylori and kidney function（UA, Cr, BUA）

**
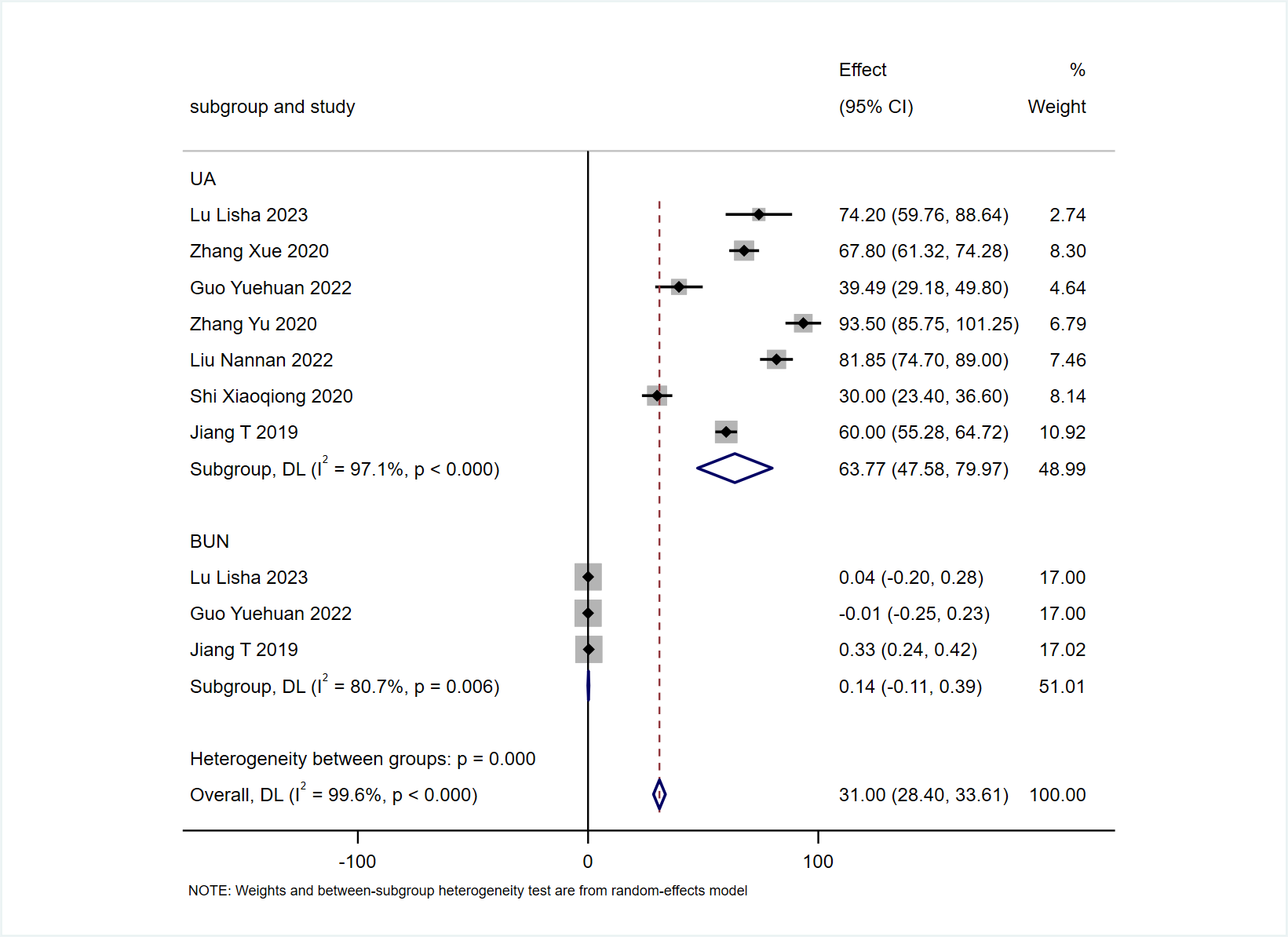
**

Supplementary Figure 8 Forest plots for NAFLD and kidney function（UA, BUA）

**
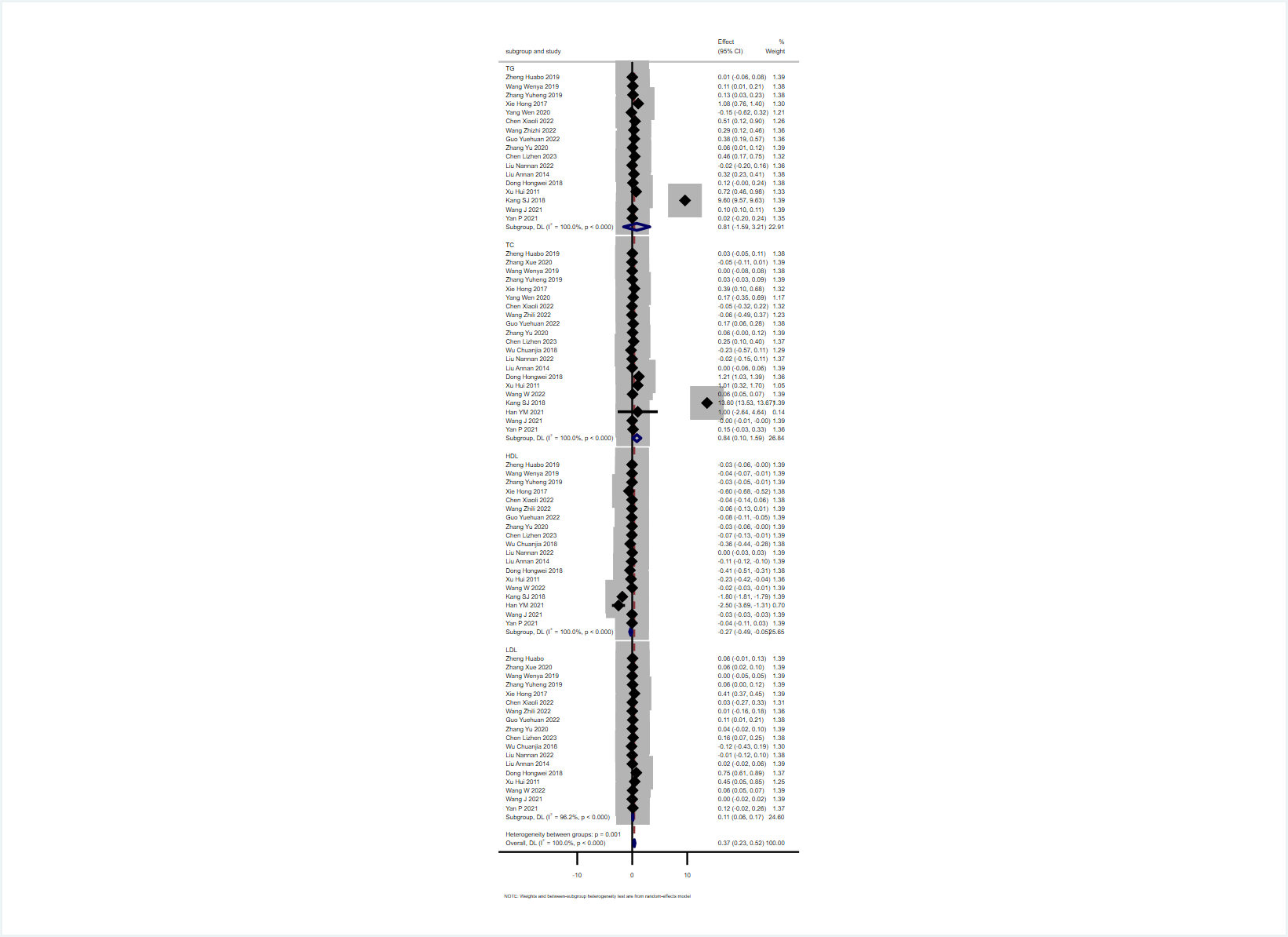
**

Supplementary Figure 9 Forest plots for H. pylori and blood lipid（TG, TC, HDL,LDL）

**
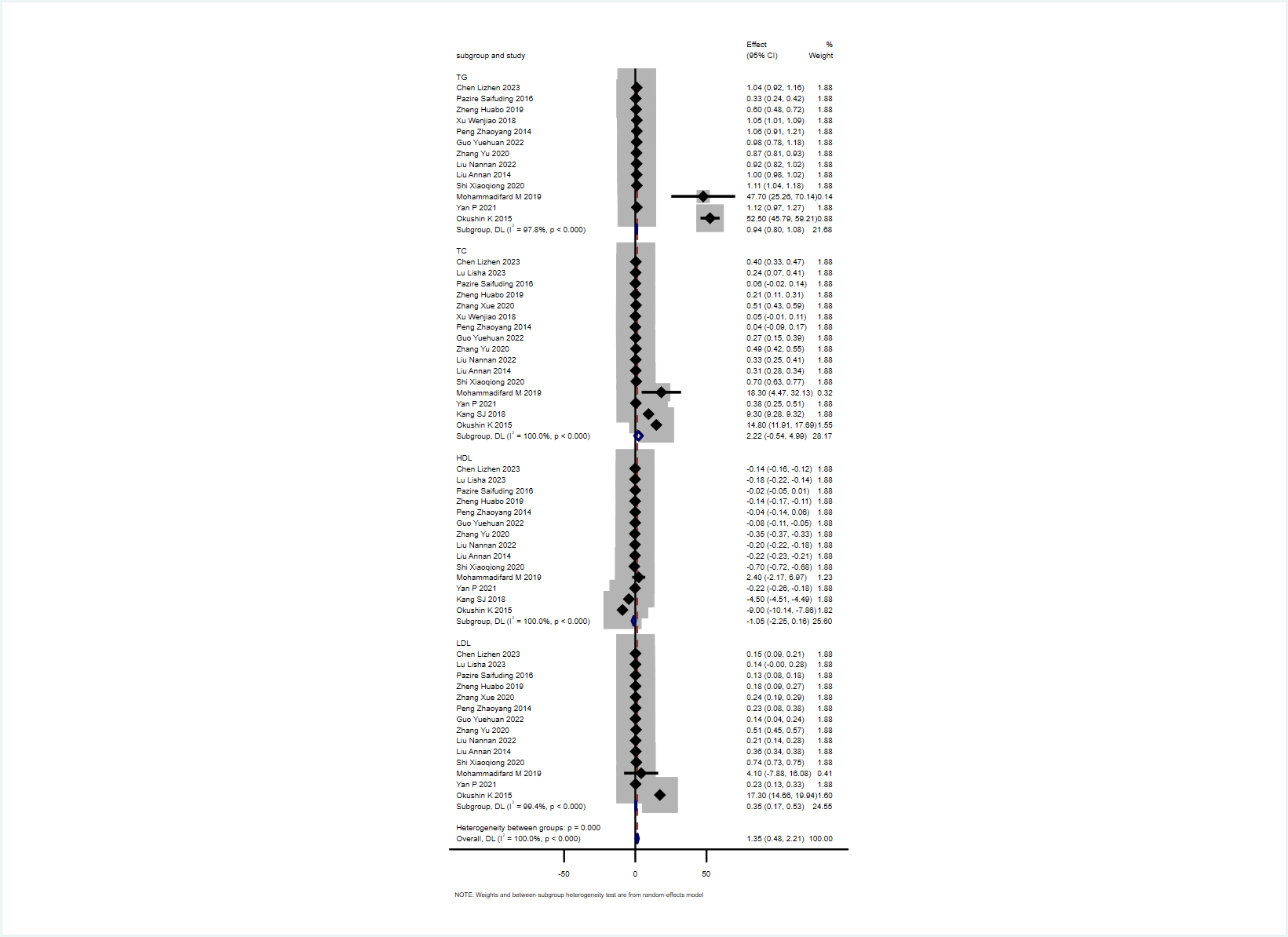
**

Supplementary Figure 10 Forest plots for NAFLD and blood lipid（TG, TC, HDL,LDL）

**
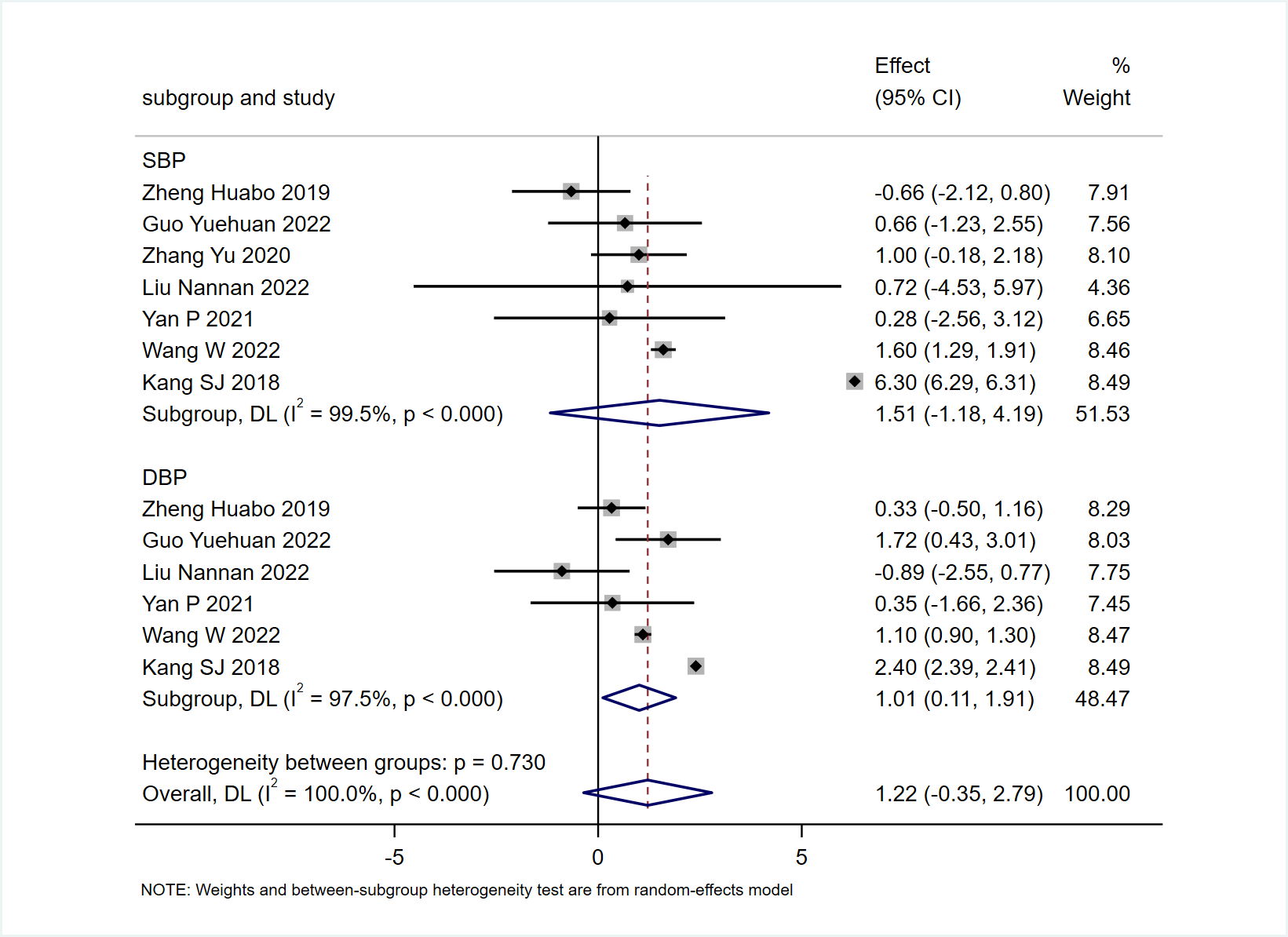
**

Supplementary Figure 11 Forest plots for H. pylori and blood pressure

**
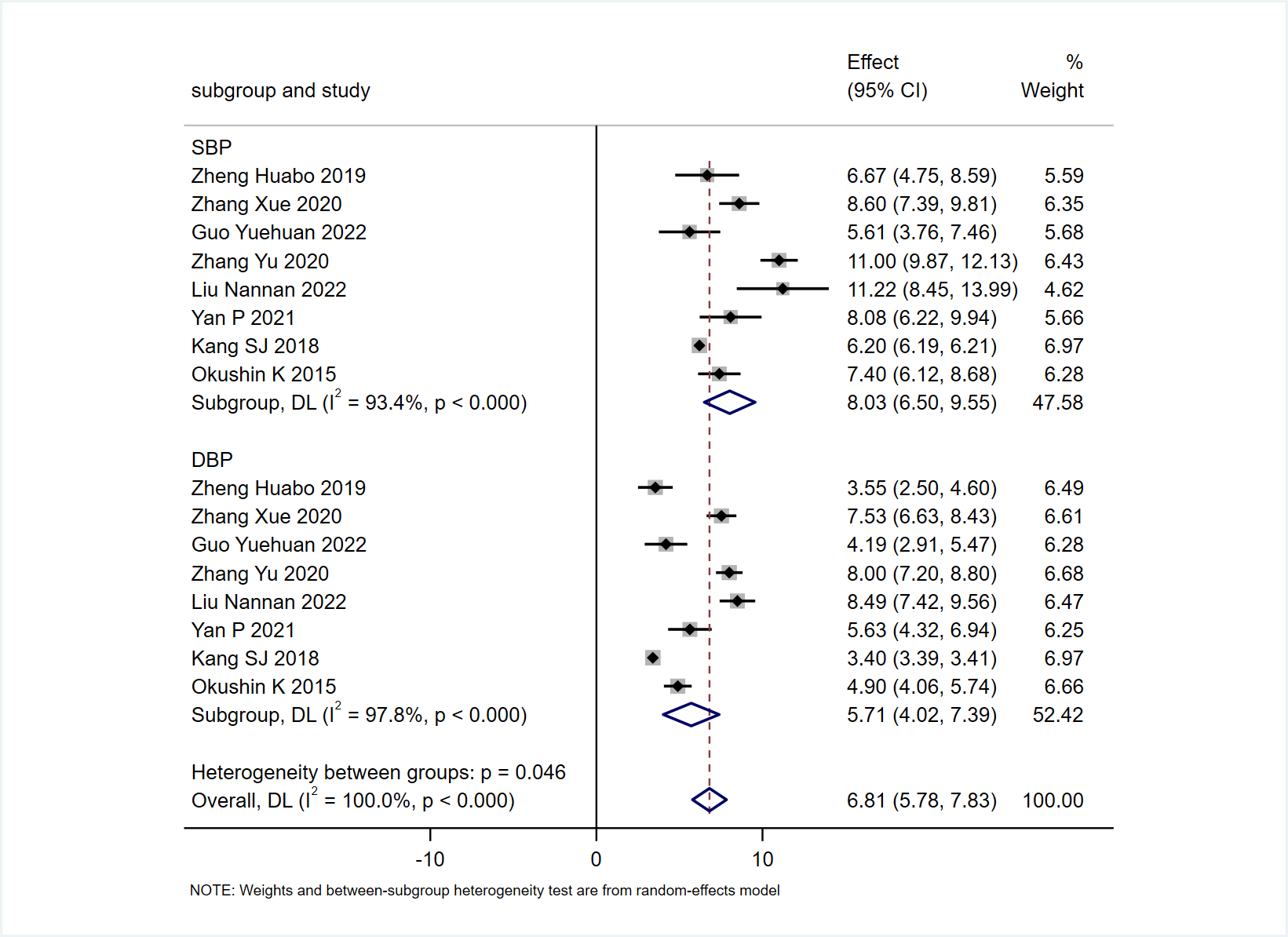
**

Supplementary Figure 12 Forest plots for NAFLD and blood pressure
